# Genetic Counseling in Huntington's Disease: Potential New Challenges on Horizon?

**DOI:** 10.3389/fneur.2019.00453

**Published:** 2019-04-30

**Authors:** Simone Migliore, Joseph Jankovic, Ferdinando Squitieri

**Affiliations:** ^1^Huntington and Rare Diseases Unit, Fondazione IRCCS Casa Sollievo Della Sofferenza Research Hospital, San Giovanni Rotondo, Italy; ^2^Department of Neurology, Parkinson's Disease Center and Movement Disorders Clinic, Baylor College of Medicine, Houston, TX, United States

**Keywords:** Huntington disease, pediatric HD, genetic counseling, intermediate alleles, neurodegenerative disease

## Abstract

Huntington's disease (HD) is a rare, hereditary, neurodegenerative and dominantly transmitted disorder affecting about 10 out of 100,000 people in Western Countries. The genetic cause is a CAG repeat expansion in the huntingtin gene (*HTT*), which is unstable and may further increase its length in subsequent generations, so called anticipation. Mutation repeat length coupled with other gene modifiers and environmental factors contribute to the age at onset in the offspring. Considering the unpredictability of age at onset and of clinical prognosis in HD, the accurate interpretation, a proper psychological support and a scientifically sound and compassionate communication of the genetic test result are crucial in the context of Good Clinical Practice and when considering further potential disease-modifying therapies. We discuss various genetic test scenarios that require a particularly careful attention in psychological and genetic counseling and expect that the counseling procedures will require a constant update.

## Introduction

Huntington's disease (HD) is a rare, hereditary, dominantly transmitted, neurodegenerative disease that leads to severe motor, cognitive, and psychiatric disability at any age, usually earlier in offspring than in their affected parent (i.e., onset anticipation phenomenon) (1, 2). The initial manifestation is typically chorea, but other movement disorders such as dystonia, myoclonus and parkinsonism may also be present (3). As the disease advances, dystonia and parkinsonism (eg., rigidity, bradykinesia) worsen the patients' functional capacity and independence and overlap to choreic movements, accompanied by gradual cognitive decline and dementia and body cachexia in the final stages of the disease.

HD is caused by an expanded and unstable CAG repeat mutation in the *huntingtin* (*HTT)* gene on chromosome 4p16.3 (4). CAG length above 35 is considered a pathogenic mutation (5) but there is a low penetrance with CAG expansions in the range 36–39. In this range the disease may not be clinically manifested until advanced age (6). On the other hand, CAG repeat instability, due to the intergenerational change of expanded number of trinucleotides, may result in highly expanded mutations beyond 60 that are highly penetrant and generally associated with young age at onset starting before age 20 (7); some patients with pediatric onset HD (PHD) may manifest a more severe HD variant and have CAG repeats >80 (2). Usually, the most extended CAG repeat expansions causing PHD in young children depend on paternal genetic transmission (2) and related germline CAG repeat instability (8).

Although a genetic test for HD is commercially available and can be ordered even before the onset of symptoms, the results are often misinterpreted and inappropriately applied in genetic counseling (9). HD is dominantly transmitted from an affected parent with each offspring having 50% chance of inheriting an unstable CAG expansion of variable repeat length in the mutated gene (10). The unexpanded repeat length in the general population is genetically influenced by specific haplotypes, with an intergenerational stepwise mechanism of CAG repeat increase, which is larger in Western than in Far East populations (11). As a direct consequence of intergenerational CAG repeat increase of borderline length alleles, i.e., the so-called intermediate alleles (IA _27−35CAG_), there are new HD mutations originating from apparently unaffected parents (10). Unstable CAG repeats are generally associated with specific *HTT* Single Nucleotide Polymorphism (SNP) haplogroups, whose frequency is higher in population of European descent and affect the prevalence of the disease (12).

Considering the unpredictability of age at onset and of clinical prognosis in HD and the many possible genetic and clinical scenarios associated with the potentially diverse inherited pre-mutational and mutational conditions, accurate interpretation and scientifically sound and compassionate communication of the genetic test result is crucial in the context of Good Clinical Practice (13). This is especially important now as new interventional trials by potentially disease-modifying therapies are on horizon (14). We review here some genetic test scenarios that, in our experience, require a particularly careful attention in psychological and genetic counseling.

## Counseling in Case of Intermediate Alleles (27–35 CAG Repeats)

Although the rate of new mutations is unpredictable, some studies postulated a frequency of about 7% of new expanded mutations originating from IAs in some populations and that the frequency of IAs influence the prevalence of HD in the general population (15). There is growing body of evidence that IAs themselves may be associated with clinical manifestations of HD, although the possibility of mild phenotypes or subclinical traits, so called endophenotypes, in some of the cases should be also considered (16). Recent evidence highlighted the association between IAs and behavioral and cognitive issues (17–21), as well as the occurrence of motor features, indistinguishable from typical HD (16). In such a scenario, the communication of the genetic test result might be problematic as it has several implications not only for the individual but also for the proband's entire family. For example, siblings of a new mutation originated from a parent carrying an IA share a risk to inherit a HD mutation themselves below 50%. Therefore, even considering the potentially high frequency of IA that may be as high as 6% of all normal alleles in some populations (16), proper counseling procedures with specific competence in clinical manifestation and genetics of HD (22), coupled with knowledge of published international guidelines (23), are critical. In families without established diagnosis of HD, DNA analysis from other members of the family are often helpful. If, for example, one of the parents or adult offsprings has an IA, this would provide support for the proband's polymorphism being “pathological,” indicating increased risk for the transmission of HD and development of symptoms in the future. It is, however, difficult to predict with any certainty the degree of risk or the age at onset of symptoms, although generally individuals with low expanded repeats have a relatively late age at onset and slow progression of the disease. Individuals carrying an IA should be carefully counseled on the potential risk of genetic transmission to children, mainly if they carry an IA_≥33_, i.e., near to the pathological edge of repeats and the highest risk to transmit new mutations (24). Pre-test counseling should therefore contemplate the increased potentiality of a large IA to expand in offspring and the current international guidelines (23) should be further updated accordingly. Furthermore, participation in registries and observational studies, such as ENROLL-HD (25), should be encouraged to clinically monitor these conditions (Table 1, Row A).

**Table 1:** Genetic counseling in Huntington disease.

**Genetic condition**	**Suggested procedure**	**Discouraged procedure**	**Comment**
A. Intermediate repeat range (27–35 CAG)	Explaining the potential risk of new mutations. Testing other members of the family.	Communicating the result as completely negative	Risk of paternal transmission of new mutations and of potential rare, subtle and unpredictable clinical presentation (10, 15, 22). Increased males' risk to transmit a full mutation to offspring (8)
B. Low penetrance range (36–39 CAG)	Confirming the presence of the mutation	Communicating the result as potentially negative	Risk of either carrying a mutation without manifesting HD (26) or to manifest HD at any age (10). Increased males' risk to transmit a full penetrance mutation to offspring (8)
C. Long expansions in pediatric Huntington's disease (>80 CAG)	Providing counseling to the whole family	Precluding any hope	The disease develops severely (2) and proper neurological and/or psychiatric evaluation and treatment are needed. Genetic test result should be communicated according to approved ethical guidelines (23)
D. HD mutation in fetus after prenatal genetic test	Ascertaining both parents' wish to interrupt the pregnancy before to carry out the test	Communicating that the disease will start late in life with no urgent consequence on the future life of the newborn	HD onset and severity are influenced by many, yet unknown, factors other than CAG repeats. No prognosis is allowed

The mechanism by which IAs result in a clinical phenotype is not well-understood but instability of CAG expansion may determine trinucleotide repeat number variability during the intergenerational parent-child mutation transmission with related somatic mosaicism, i.e., cell populations carrying different expanded repeat lengths in several body tissues (27), especially in differentiated neurons (e.g., in the striatal medium spiny neurons or cortex) (28), which may explain this phenomenon (10, 29). There is already evidence of increased mosaicism of the full CAG mutation in brain striatum neurons of transgenic mice and of symptomatic patients with large size mutations in the post-mortem neuropathological brain sample (28, 30). Therefore, we cannot exclude that CAG instability of IAs may play a key role in determining somatic triplet mosaicism, some cell populations having full mutations in the central nervous system. In other words, subjects carrying IAs in their blood cells might theoretically have a mosaic of CAG lengths, including full mutations in their brain cells and this phenomenon might contribute to symptoms of HD or endophenotypes. Future studies on peripheral, easy-to access, tissues, e.g., blood, buccal, sperm cells, might offer new chances to investigate potential markers of CAG mosaicism. In this regard, genes or their products that either facilitate somatic expansion or increase CAG mosaicism in different body tissues, may represent additional therapeutic targets (2, 31).

## Counseling in Case of Low Penetrance Alleles (36–39 CAG Repeats)

When the polymorphic CAG stretch is longer than 35, the disease may develop at any age with tendency to anticipate before the affected parent's onset age. Allele repeat numbers included in 36–39 CAG length are considered low penetrance mutations (6, 10) and may randomly occur in the general population with an estimated frequency of 1/400 (26). In most of these cases, subjects do not show full HD phenotype (or will show some clinical symptoms sometime at advanced age). These conditions are difficult to detect, unless people with no evidence of positive family history show typical symptoms of HD. Considering the subtle and non-specific clinical manifestations and the possible negative or misinterpreted family history, most of these situations remain undiagnosed, thus contributing to the underestimate of the disease frequency. Communication of a genetic result when the mutation is in the low penetrance range is problematic because it may never be associated with development of a clinical manifestation of disease. (Table 1, Row B). There are many factors that must be considered during genetic counseling in these individuals, including penetrance variability determining age at onset, increased longevity in aging general population (32), parent-to-child onset anticipation with the offspring exhibiting symptoms at an earlier age than the parents, and even non-genetic factors (33) (Table 1, Row B). However, the parent-child transmission of a low penetrance mutation may cause an increased number of repeats in offspring. Considering the male germline susceptibility to CAG instability (24) and a non-negligible rate of silent low penetrance alleles in the general population (26), pre-test counseling sessions as well as a further update of the international ethical guidelines should address the potentiality of the magnitude of the risk of expansion for future generations.

However, many other environmental and genetic factors, other than the expanded CAG repeats, may contribute to explain the penetrance of the mutation as well as of IAs, and their effects on HD development (31, 34, 35). These, yet still far to be fully elucidated factors, will certainly offer additional clues and discussion to counseling guidelines in future.

## Counseling in Case of Markedly Expanded Alleles (> 80 CAG Repeats)

CAG repeat expansions may sometimes greatly increase during the intergenerational parent-child transmission, sometimes reaching over 100 CAG repeats, resulting in PHD with onset before the age 12 (2, 10, 36, 37). PHD children with large-sized and somatically unstable mutations beyond 80 CAG repeats may be characterized by atypical clinical manifestations at onset and overtime, compared to the adult-onset variant and to other juvenile patients with relatively shorter repeats, mental and motor developmental delay, predominant gait disturbance and dystonia, seizures, reduced life span, and more-rapid motor progression (2). The interpretation of the genetic test in case of children should always take into account the international guidelines for testing minors (38) and include counseling of both parents whenever possible. Because the most frequent transmitting parent is the father in case of PHD with large expanded repeats, psychological counseling should take into account the distress not only to the affected individual and the affected parent, but also the caregiver (in this case the mother more often than the father). The genetic test result may, therefore, have a profound impact on the future life of all family members and need to be delivered carefully and compassionately (22) (Table 1, Row C). The interpretation of the most common CAG repeat expansions (e.g., 40–50 CAG) in young adults or minors with psychiatric, but no motor symptoms, is also challenging as the course of the disease is difficult to predict, although the age at onset and the progression often mirrors those in other, affected, adult members of the family (2). These cases will deserve additional in deep analysis for it concerns clinical and biological implications, psychological and genetic counseling management and therapeutic strategies.

## Counseling in Case of Subjects Homozygous for CAG Repeat Expansions

This condition occurs very rarely in a dominant disease and is due to the intermarriage of two subjects heterozygous for expanded CAG repeat mutation. In this case subjects inherit two mutations (i.e., one from each mutation carrier parent). Reports of homozygous cases have described relatively frequent occurrence of intermarriages among consanguineous relatives in certain populations with high density of HD (i.e., the large Venezuelan kindred) (34, 39). Such rare condition does not seem to affect age at onset more severely than a heterozygous condition, due to the fully dominant, gain-of-function, effect of the mutation (40). Whether it affects the clinical presentation (i.e., more frequent atypical presentation than heterozygous patients), the progression of HD course, or both (39), still needs to be explored in larger cohorts. However, independent of the phenotype, subjects who carry two expanded mutations always transmit a CAG repeat expansion to their offspring whose risk of inheriting HD becomes of 100%, instead of the typical 50% (41). There are at least two main issues related to such particularly unfortunate condition. (1). How to communicate to at-risk offsprings that they will certainly inherit a HD mutation? (2). How to protect the privacy of the at-risk offspring, considering that the genetic result of their affected parent would unequivocally disclose their own risk to inherit HD mutation, independently from whether they request a genetic test or not? Caution is therefore needed by counselors in these cases and a further update of the international ethical guidelines should address such a rare occurrence (23).

## Prenatal Counseling

Genetic predictive counseling should include the 5Cs: Consent, Counseling, Confidentiality, Cost, and Consequences. Prenatal counseling represents a unique example how advances in genetic and *in-utero* techniques must be translated into genetic counseling based on ethical and scientific principles before any important decision is considered, including the interruption of pregnancy (42). Reproductive choices for at risk individuals include: (i) the wish to have a pregnancy free of HD and without the risk of a mutation carrier offspring; (ii) the decision to test an ongoing pregnancy by chorionic villous sampling, which may be performed at 8–10 weeks after conception (the sample obtained from chorionic villi can be tested), with the option of termination of the pregnancy if the fetus carries the HD mutation; (iii) the decision to eventually perform the predictive testing before requesting the prenatal diagnosis (by contrast, in case of positive result in the fetus before the at risk parent undergoes the presymptomatic test, the genetic status of the at-risk parent would be indirectly revealed); (iv) the request of pre-implantation genetic diagnosis (PGD) that is performed as part of an *in vitro* fertilization (IVF) procedure at specialized IVF centers (42). The counselor should discuss each of these options so that the at risk couple has a chance to make the most informed reproductive decision since the pre-pregnancy stage, in agreement with recently reports (43). Clear and unambiguous communication, as well as a proper psychological support, are essential features to prevent any misunderstanding (Table 1, Row D). Unfortunately, miscommunication during counseling may result in unexpected outcome and may risk to lead to wrong decision makings by parents.

There are many ethical issues that must be considered before performing prenatal testing in an attempt to prevent HD. These include variability of HD mutation penetrance and the role of potential modifiers acting to modulate onset and progression severity of the disease. The understanding of natural history of HD is improving as a result of many published worldwide observational studies (2, 25). Accordingly, the genetic and psychological counseling should be performed by professionals who are well-informed about and experienced in HD.

## Counseling and HD Family Organizations

One of the most challenging issues for all HD symptomatic and at-risk persons is the profound denial of the presence of HD in the family and discrimination and shunning of the affected individuals from their own family and from the society. In the United States, the Genetic Information Non-discrimination Act (GINA), enacted in 2008, was an important step toward preventing discrimination based on diagnosis of a neurodegenerative disease, such as HD. This act forbids employers from denying employment, promotions, or health insurance coverage to people when genetic tests show they have predisposition to an inherited disease. It also forbids health insurance companies to use genetic tests to determine an applicant's risk profile. Unfortunately, the act does not apply to life, disability, and long-term care insurance.

Lay organizations and support groups play a crucial role not only in creating bridges between scientists and patients, but also in spreading important information among families, including the importance of following the proper “procedure” if and when somebody discover or want to discover if he/she is gene positive. Moreover, they can also play a role—together with researchers and clinicians—in motivating patients and relatives to participate into research programs, i.e., clinical observational studies (i.e., ENROLL-HD) and therapeutic trials. HD Cope is an international coalition connecting three umbrella organizations: the HD Society of America (HDSA), the European Huntington Association (EHA), and the Huntington Society of Canada (HSC). These and other initiatives and organizations can play a critical role in improving quality of life of HD patients and their families.

## Conclusion and Recommendations

In this review we attempt to summarize current knowledge about the HD mutation and how information about CAG repeats in the *HTT* gene can be used to provide sound genetic counseling, which can possibly extend to other neurodegenerative diseases (44). By involving families in observational (i.e., ENROLL-HD platform) and interventional trials, clinicians have an opportunity to include adult patients and their relatives (e.g., partners, symptomatic minors, premanifest adults) in research-based networks that provide current knowledge about developments in genetic testing and novel therapies in HD (45).

With advances in research and improved understanding of mechanisms of dysfunction, degeneration and abnormal development of brain in HD, innovative experimental therapies, such as antisense oligonucleotide (ASO), gene editing by CRSPR/CAS, or suppression of gene modifiers of age at onset may, hopefully, be transferred from basic research into clinics in a not so far future (14, 46, 47). Theoretically, such strategies will consequently require new competencies in the pre- and post-test counseling as well as in pre- and post-conceptional approaches. Moreover, we need to remind that we still need to fully understand how HD develops and why there is such a wide spectrum of heterogeneous clinical manifestations among patients. Accordingly, new potential challenges in genetic diagnosis will require further and constant updates of guidelines and the psychological support should be carefully offered during all counseling procedures.

## Author Contributions

SM contributed to manuscript preparation, review, and critique to the project. JJ contributed to design, conception, organization, design, review, and critique to the project. FS contributed to design, conception, organization, execution, design, review and Critique to the project, and wrote the first draft.

### Conflict of Interest Statement

The authors declare that the research was conducted in the absence of any commercial or financial relationships that could be construed as a potential conflict of interest.
